# Developmental Considerations in Obsessive Compulsive Disorder: Comparing Pediatric and Adult-Onset Cases

**DOI:** 10.3389/fpsyt.2021.678538

**Published:** 2021-06-14

**Authors:** Daniel A. Geller, Saffron Homayoun, Gabrielle Johnson

**Affiliations:** ^1^Pediatric OCD and Tic Disorder Program, Department of Psychiatry, Massachusetts General Hospital, Boston, MA, United States; ^2^Harvard Medical School, Boston, MA, United States; ^3^Psychiatry and Neuroimmunology Program, Department of Psychiatry, Massachusetts General Hospital, Boston, MA, United States

**Keywords:** obsessive compulsive disorder, pediatric, child and adolescent, developmental, neuropsychology, immune, inflammation, neuroimaging

## Abstract

There appear to be two peaks of incidence of Obsessive Compulsive Disorder (OCD), one with a pre-adolescent onset and another in early adulthood. As new cases are added, the cumulative prevalence of OCD increases, but the great majority of cases have an onset in youth. The notion that early onset OCD represents a unique developmental subtype of the disorder has been considered by many researchers based on several specific age-related factors. Ascertainment and early intervention in affected youth is critical to abbreviate the functional impairments associated with untreated illness. In this paper we review the clinical, familial and translational biomarker correlates seen in early onset OCD that support the notion of a developmental subtype and discuss implications for research and treatment aimed at this cohort. The importance of cognitive, academic and social development tasks of childhood and adolescence, illness-specific and familial factors, and immune-mediated inflammatory factors are discussed, with their implications for management.

## Introduction

For decades, clinical research has posited a developmental subtype of Obsessive Compulsive Disorder (OCD) that affects youth, and which may be distinct in important ways from the adult-onset form. Evidence for such a developmental subtype draws from multiple lines of observation and investigation at the clinical, translational and basic science levels. Despite this, the latest incarnation of the Diagnostic and Statistical Manual of the American Psychiatric Association, the DSM5 (1) does not specify a developmental subtype, but rather includes two different “specifiers” that apply particularly to children and adolescents. In this review, we will examine the differences between the early- or pediatric-onset form of OCD (these terms are used interchangeably) and the adult-onset form, including epidemiology, symptom presentations, clinical correlates, comorbid disorders, familial and genetic factors, environmental and epigenetic factors, salient neurocircuitry, treatment response, course and outcome. Many of these features are different in youth with OCD compared to adult OCD subjects.

Children and adolescents generally display a pre-pubertal onset of their symptoms, some as young as 6 years of age, and may show a distinct symptom pattern (2–4) as well as distinct array of concurrent psychopathology (2) and neuropsychological function (5, 6). Familial loading (7–9), and the role of the family (10) are amplified in pediatric cases, and youth may be susceptible to environmental triggers that are notably present in the early years (11, 12), and may display unique biosignatures (13). Outcomes are often more favorable for pediatric OCD and treatment response is robust and more durable (10).

The recognition and management of OCD in youth generally require specialist knowledge and care, not least because of the numerous and specific developmental tasks and milestones of early life that may be disrupted by illness, with potential long term adverse consequences (14). For this reason, OCD affecting youth and more especially, untreated or inadequately treated illness, is of particular concern to OCD clinicians and researchers. Although the effects of early intervention to mitigate long-term adverse outcomes has not been systematically studied, these considerations strongly support effective early intervention in youth affected by OCD (14).

## OCD in children, adolescents, and adults

### Definition in DSM 5

While the core diagnostic features of OCD are the same across the lifespan, the DSM5 includes two “specifiers” especially relevant to pediatric cases (Table 1). Core symptoms include intrusive obsessions and compulsions (worries and rituals) that are time-consuming, distressing and functionally impairing, that are not better explained by the physiological effects of a substance or another mental disorder.

**Table 1:** DSM5 OCD specifiers relevant to pediatric OCD.

Specify if:	With good or fair insight: The individual recognizes that obsessive-compulsive disorder beliefs are definitely or probably not true or that they may or may not be true.
	With poor insight: The individual thinks obsessive-compulsive disorder beliefs are probably true.
	With absent insight/delusional beliefs: The individual is completely convinced that obsessive-compulsive disorder beliefs are true.
Specify if:	Tic-related: The individual has a current or past history of a tic disorder.

#### Definition of Early- or Pediatric-Onset OCD

Pediatric OCD is defined as onset before age 18 years of age. The first comprehensive description of a case series was published in 1991 in (15) “The boy who couldn't stop washing: The experience and treatment of obsessive compulsive disorder,” demonstrating that the recognition of this disorder in youth is relatively recent.

### Epidemiology

OCD in children may go unrecognized for some time and in several of the epidemiological studies, youth identified with the disorder had usually not come to clinical attention or received a formal diagnosis (16, 17). Reasons for under-recognition include the limited verbal skills of younger subjects who may either be unable to articulate their intrusive thoughts, or not recognize them as irrational. Even in those with moderate insight, symptoms are often secretive and hidden due to embarrassment or shame. Sometimes impairments are seen only in more familiar home environments, may be domain specific, and may be masked in more public settings such as school. In addition, some symptoms, such as perfectionism, are unwittingly reinforced by parents and teachers based on good grades. Browne et al. (18) reported a cohort prevalence of 0.84% in a recent epidemiological study using the Danish health registry of more than one million youth. This prevalence is likely the most accurate to date and falls between the earlier estimates of 1–2% (16) and the 0.25% point prevalence reported by Heyman et al. (17). The prevalence of OCD in adult populations has been variously reported between 1 and 3% (19), but these figures may include some subthreshold cases. Although one would expect an increasing cumulative prevalence of cases (and therefore a higher cumulative prevalence) in adults as new cases are added to the affected population, the relatively higher rates of remission (10) (~one third to one half remit or improve to subthreshold levels) of pediatric cases offsets the new adult incidence so that the overall prevalence does not change much over time. The prevalence of both adult (20) and pediatric OCD (18) has been noted to be remarkedly consistent across different counties and continents.

### Clinical Features

#### Age at Onset

**Figure 1:**
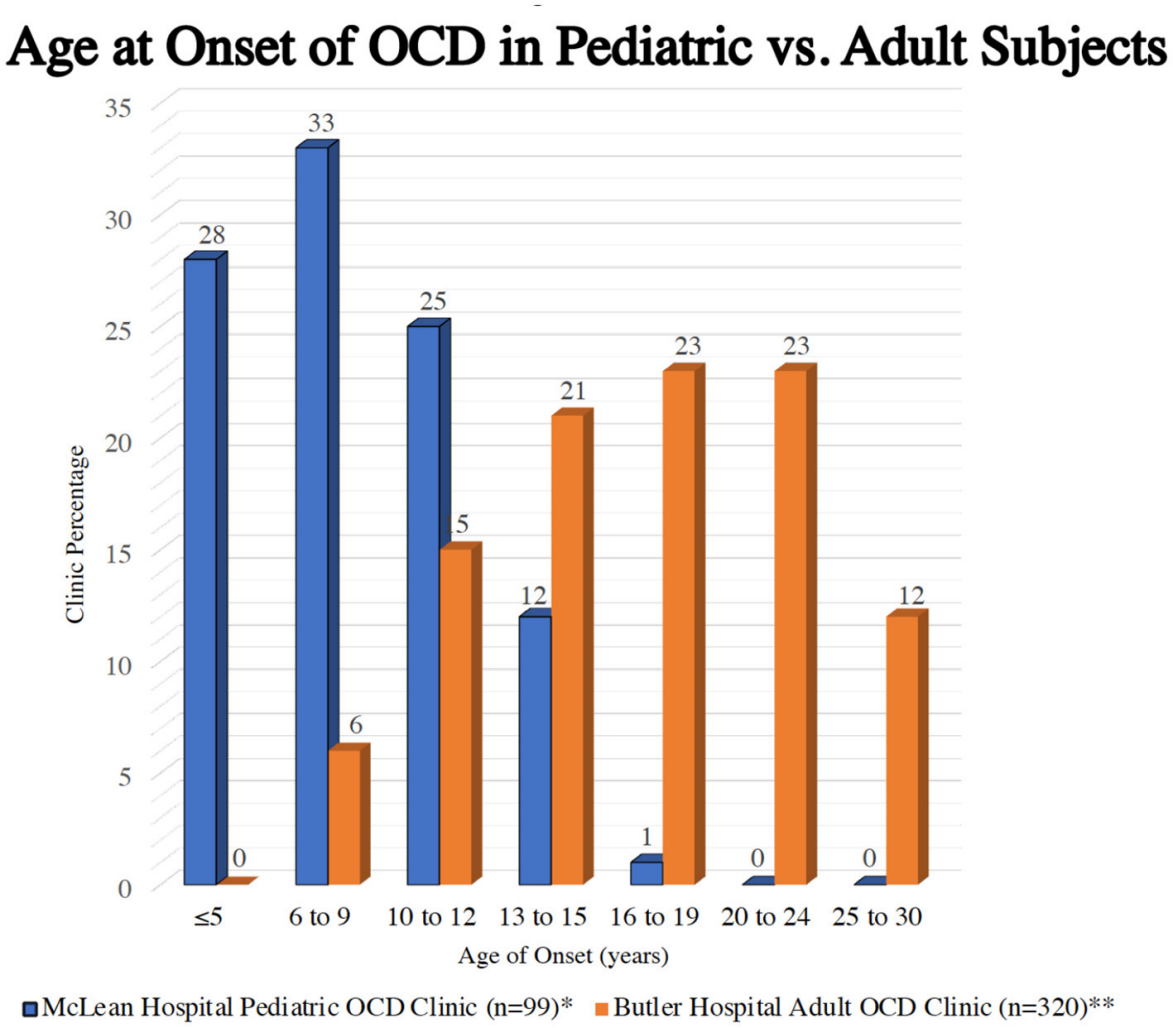
A Bimodal distribution of incidence of OCD across the lifespan. ^*^Geller et al. (22). ^**^Rasmuseen et al. (23).

It is notable that there are two peaks of incidence (new onsets) of OCD, one early peak with a mean age of 9 to 10 (with an SD of ± 2.5 years) years of age and by definition, pre-pubertal, so that two thirds of affected youth will have an onset between about 7 and 12 years of age and well-before adulthood (21). There is a second peak of incidence in the early 20's (see Figure 1) (22, 23), but, because of cumulative prevalence as new cases are added to the affected population starting with the early peak, two thirds of current adult cases have their onset before adulthood, a potentially confusing finding (24).

As noted above, there is often a lag between age at ascertainment and age at onset due to under-recognition (25), a finding that may have clinical consequences. Because age at ascertainment generally lags behind onset by several years, onsets are acquired by anamnestic parental report and may therefore be inexact. We may look to other areas of medicine for an understanding of distinct peaks (but overlapping curves) of illness onset. In diabetes, the phenotype of high blood sugar, glucosuria and ketonuria are well-known, but it was only in the era of modern medicine that the distinction between type 1 diabetes (pancreatic insulin insufficiency resulting from auto-immune islet cell destruction) and type 2 diabetes (peripheral insulin resistance from persistent high circulating insulin in overweight subjects) was understood. Type 1 is known to affect younger people with a typical age at onset that is less than type 2, which more often affects older subjects related to insulin resistance secondary to obesity (26). However, the recent increase in childhood obesity in the Western world has led to higher incidence of type 2 diabetes in youth blurring these boundaries (27). Thus, similar phenotype does not imply a single etiology or set of genetic risk factors, even when some common pathophysiological pathways are involved.

#### Gender Ratio

There is some uncertainty about this issue. Whilst earlier reports generally found a male predominance (25, 28), the more recent Danish epidemiological survey (18) reported a slight female preponderance in youth with OCD (29) similar that that generally reported in adult cohorts (30) Notably, common comorbid conditions that are frequently seen in younger OCD subjects, such as chronic tic disorders and Tourette's syndrome, ADHD, and autism spectrum disorders (ASD) all show a clear male preponderance. The weight of evidence from clinical samples suggests that pediatric OCD does indeed represent a developmental subtype, with male preponderant cases with concurrent tics, ADHD and ASD-like psychopathology that often remit in adolescence and constitute one of the core hallmarks of such a subtype. Rates of OCD may also be much higher in transgender populations, with up to 9.8% prevalence estimated for transgender women and 7.6% for transgender men (31).

#### Primary Symptoms

##### Normal Development

Typically, preschool-age children engage in ritualistic behavior, for example, routines at bedtime or mealtime, but these provide familiarity and comfort and are not disruptive. These are usually easily managed within normal family structure and are not disruptive to either child or family. However, for some children, insistence on routines or rules shows a high degree of inflexibility and rigidity and when not complied with, can lead to behavioral outbursts and disrupted family function. It has been reported however that these children's insistence on excessive rituals may be a red flag and indicator of risk for OCD in later childhood (32). Magical thinking, or the belief that a person's thoughts or actions can somehow influence real outcomes even though there is no causal connection between these, reflects normal development in very young children. This ego-centric world view ascribes influence to (irrational) rituals that are often performed to avert feared bad or unsafe outcomes. Normal cognitive development is associated with more reality-based apprehension of causality by the time most children show an onset of OCD (age 8 years and older), so that persistent magical thinking at this age is not normal. It should be noted that magical thinking can persist and be seen in adults, but there is some overlap with extreme superstitions or those that are heavily culturally based. Collecting and saving of personally meaningful items such as sports cards, coins, stamps, comic books etc., is also normal behavior in youth and should not be confused with hoarding of items of little value such as bits of lint, old bottle tops or pieces of paper that lead to clutter and refusal to discard without family conflict.

##### Pediatric OCD Symptoms

In addition to a diminished capacity to articulate their concerns, some younger subjects may lack the ego function to recognize their obsessions as abnormal. On occasion, obsessions must be inferred by the parents who observe rituals in their children (33).

Obsessional anxiety frequently contains themes that reflect exaggerated developmental concerns at any given age, which may be difficult to dissociate from normal childhood development (22). For example, young children may struggle with increasing autonomy and independence, especially around separations from important parental figures, leading to intrusive fears of harm or loss of attachment figures. While this may appear as more typical separation anxiety, checking behaviors and magical rituals are also common (34). Recurrent worries about catastrophic family events or loss can also appear in youth with OCD with no premorbid history of separation anxiety disorder. Verbal checking and reassurance seeking often inadvertently engage parents in accommodation behaviors. Hoarding, saving and collecting rituals affect up to a quarter of youth with OCD (35) and are excessive, often with items that are unusual for the age, causing clutter and upset if discarded. Youth with hoarding also display other rituals and show increased rates of tic disorders. In adults, hoarding has typically been associated with poorer exposure and response prevention (ERP) outcomes but in a recent study, CBT treatment response in youth who hoarded was not adversely impacted (35). Symmetry and ordering, as well as “just-right” rituals have been reported to be more prevalent in pediatric OCD (36), and may reflect comorbidity with chronic tic disorders. Some children cannot articulate specific cognitions that drive rituals, reporting instead a vague feeling of unease or discomfort until certain actions are performed repeatedly. Without concrete cognitive obsessions, ERP may be less successful because habituation to vague but intense feelings of unease cannot utilize a cognitive strategy to “boss back” the urge to ritualize. For example, discomfort from the sensation that one's hands are greasy may be more difficult to reason through than the idea that they may have touched an object with dangerous germs.

Adolescents often experience tensions around sexual, moral and religious ideas and these thoughts are more often prevalent in the obsessional content of adolescent patients at an age in normal development when such concerns are more likely to cause anxiety or conflict (36). Scrupulosity is therefore seen more commonly in adolescents and young adults, leading to confessing and apologizing rituals (37). The role of religious authorities in management is to be considered for these patients.

Most youth report contamination obsessions at some time, similar to adults, and consequently display washing, cleaning and avoidance rituals, but, similar to adults, the primary associated affect may be disgust (“gross”) rather than fear-based, for example, of germs or harm. Most will also demonstrate obsessions and compulsion from more than one “category” and gender has not been reported to influence specific symptoms. Finally, while some OCD symptoms tend to persist, their relative presence and interference may change with time showing less stability over time than symptoms in adult patients (38). Factor or cluster analysis has often been used to better identify subtypes of “dimensions” of OCD (3) and there is a “dimensional” form of the Yale-Brown OCD Scale DY-BOCS) (39) but this approach has not shown any consistent benefit for genetic or translational approaches or yielded particular biological signatures. More recent network analysis of symptoms to identify meaningful symptom structures may however prove more useful for subtyping subjects for both treatment trials and further translational investigation (40).

While not a core symptom of OCD, children with this disorder also frequently display irritable behavior, that may in turn be a cause of greater impairment of function, especially in the domain of the family (41). Storch et al. (41) also reported that profound irritability and tantrums led to more parental accommodation (in order to manage conflict), which is known to reinforce OCD behaviors. According to Guzick et al. (42), parents often report irritability in their affected children, driven by anxiety when frustrated by a need for perfection or certainty, or by an overestimated assessment of responsibility or threat. These findings in youth also conform to reports of greater distress, impairment and treatment resistance in OCD sufferers in the extant literature (43–46). Treatment protocols may benefit from taking the high levels of irritability into account when designing CBT interventions for some youth (47) requiring close work with families (48).

#### Insight

Insight in youth with OCD may be limited. Selles et al. (49) reported a meta-analysis of 573 children and adolescents enrolled in several North American and international CBT treatment trials and found that only 63% had good or excellent insight. Similarly, adults with the condition have been found to have poor/no insight in 13.8–30.7% of cases (50). The construct of insight may be difficult to measure quantitatively in youth with OCD as shown in a study that found no direct correlation between insight and treatment response, but at the same time, found that youth who had limited appreciation of the impairment from their OCD and greater avoidance behaviors, showed less likelihood of response to ERP (49), an apparent contradiction. Insight likely varies with anxiety levels and cognitive maturation, rather than being a static quantity. Studies of adults with OCD may be more informative regarding the adverse impact of insight on treatment. A number of adult studies (23–26) suggest a correlation between (poorer) insight and (poorer) outcome with standard of care treatment but insight at baseline, and changes in insight with CBT, show contradictory results (27, 28). One pediatric study (29) suggested that poorer treatment response correlated with less insight at baseline across all treatment modalities (30). Frank delusional beliefs and psychosis are very uncommon in pediatric OCD, although schizophreniform illness may first manifest as obsessional anxiety.

### Comorbidity

All studies, including epidemiological studies of non-referred cases (16), find that most OCD-afflicted youth will have concurrent psychopathology, especially over time. Clinically referred and ascertained cases have even higher rates of comorbidity, as high as 80% (51). There is an ontogeny of comorbid conditions affecting those with pediatric OCD that is distinct for youth compared with adult-onset cases (51). This means that certain comorbid conditions arise at different and specific ages over time. As with adults, mood and other anxiety disorders are very common, but some frequently seen concurrent disorders are classically pediatric-onset disorders, such as Attention Deficit Hyperactivity Disorder (ADHD) and Tourette's Disorder (TD), and predominate in pediatric cases. The majority of children with ADHD and tic disorders are male, and they typically have an earlier onset (2). If gender ratios averaged across all the pediatric years indeed show equal prevalence, as noted above, it would suggest that older affected youth trend toward a female preponderance with less comorbid ADHD and tic disorders. The triad of OCD, Tourette's disorder and ADHD is not uncommon in pediatric cases, and reflects an underlying inhibitory deficit affecting thoughts (obsessions), motor behavior (tics) and attention (52). Poor inhibitory control may be identified by deficits in neuropsychological tests of inhibition in affected families (53). Neural maturation often brings improvement or remission of some of these symptoms with diminished tics and improved executive function (2). Comorbid mood and anxiety disorders occur at all ages and may persist into the adult years, sometimes becoming the main concern. As described above, youth with predominant irritable presentations may also meet criteria for oppositional defiant disorder (ODD) or even disruptive mood dysregulation disorder (ICD10 F34.81) (54).

One of the more difficult diagnostic dilemmas occurs when there is some evidence of an autism spectrum disorder (ASD), which shows an infrequent but notable comorbidity with pediatric OCD (51) and is thus distinguished from adult OCD. This overlap presents challenges for both diagnosis and treatment and has a major impact on treatment and educational interventions, role of the family, and outcome (55). Defining symptoms of ASD such as a restricted and narrow range of interests and activities, and stereotypic and repetitive behaviors can lead to confusion in younger children. It is estimated that perhaps 5% of OCD-affected youth also meet the diagnostic threshold for ASD (51). Helpful considerations are whether symptoms are subjectively experienced as ego-dystonic (OCD) or ego-syntonic (ASD), whether anxiety drives rituals (OCD) or occurs when rituals are impeded (ASD), whether rituals are resisted (OCD) or preferred, self-stimulating, and providing gratification (ASD). When classical OCD symptoms such as washing or checking are present, OCD can be reasonably inferred.

The presence of comorbid disorders may speak to a developmental subtype of OCD with conditions unique to pediatric cases, but also has relevance to phenotype, treatment and outcome (see Treatments section below). The DSM 5 specifier, “with tics” is a clear acknowledgment of this with a preponderance of “just right” rituals that may be confused with complex tics (recurrent touching or tapping) (56). Furthermore, certain comorbid disorders have been shown to diminish treatment response, both to conventional CBT (57) and also to standard SSRI treatment (58). Geller et al. (58) also reported that relapse of OCD was more common following discontinuation of paroxetine in a placebo-controlled randomized withdrawal trial in those with more comorbid conditions.

## Familial and genetic factors

### Role of the Family

Children are embedded in family units and not surprisingly, parents are often deeply engaged in behavior that accommodates their child's distress that, by providing relief in the immediate moment, inadvertently reinforces the cycle of obsessions and compulsions (59–62). Verbal reassurance, engaging in back and forth verbal rituals, and performing actions that permit children to avoid feared stimuli are all quite common. Examples include opening doors, excessive laundering of “contaminated” personal items such as clothing or linen, or arranging meals in a highly ritualized fashion (63). Add to this the common occurrence of anxiety or even OCD in a parent, given the highly familial nature of this disorder (64), and the management can become complex. Family members, including siblings (65), therefore play a central role in both the maintenance of OCD symptoms and by extension, the effectiveness of CBT, in order to allow response prevention to occur. Scales to assess and quantify the degree of family accommodation (FA) such as the Family Accommodation Scale for OCD–Interviewer Rated [FAS-IR] (66), may be useful and can show decreasing scores that reflect improvement over time with standard CBT protocols.

Some treatment intervention models such as the Pediatric Obsessive Compulsive Treatment Study for Young Children [POTS Jr] (67) specifically incorporate structured approaches for family involvement to address unhelpful accommodation. A recent rigorous randomized controlled multi-site trial found that impairments in social, home, and school life were significantly correlated with the degree of FA at baseline, but notably, in this study, baseline FA did not predict a poorer outcome over the course of treatment. Family accommodation decreased significantly with successful implementation of CBT and treatment response, with gains maintained at 6 months follow-up (68) in youth with OCD. Scalar FA scores fell by more than half over a 10 session CBT protocol to non-clinical levels (69). In this cohort, the relationship between severity of OCD symptoms and functional impairment was mediated by FA. In other words, greater involvement of family members was associated with worse OCD symptoms and worse illness-associated impairment (68).

The impact of FA can be widely felt within families of both children and adults with OCD, although there tends to be less direct involement in rituals by relatives of adult patients compared to parents of children with the condition (70). Storch et al. (71) reported that family members often took over responsibilities of affected youth, while Peris et al. (61) reported that conflict within families increased with the degree of FA. The ability of youth to tolerate exposures delivered as part of CBT may also be diminished by high levels of FA (72) and the outcome of such treatment may be adversely affected (69, 71, 73, 74). For this reason, effective treatment often underscores the need to recognize and manage FA (51, 67, 75–77).

### Genetics

It should be clear from the above text that not all that is familial is also genetic. Disentangling familial environmental effects from genetic contribution requires segregation analyses of twin and family genetic studies, as well as the more recent genome-wide association studies (GWAS) (24, 64, 78). Overall, heritability estimates for OCD are in the range of 0.25–0.28 (78). However, when an index case is a child, that is, an pediatric case, the risk of OCD in a first-degree relative is approximately two-fold (79) and as high as 26% compared to about 12% risk in adult-onset cases (64). This means that a pediatric disorder is likely the result of a higher cumulative genetic loading of many genes of small effect. Recent GWAS studies have lent support to the notion that, like most psychiatric disorders, OCD is a polygenic disorder (80), with genes implicated in serotonin transmission (81) and glutamate pathways (9, 78, 82) at the very least. In a recent report from the cross-disorder group of the psychiatric genomics consortium (80) substantial pleiotropy of genetic loci was identified across eight psychiatric disorders. The strongest correlations with OCD were with anorexia nervosa and Tourette's disorder (but not ADHD) (80). Copy number variants (CNVs), which describe large mega-base deletions or duplications have also been implicated (13). Genetic studies of pediatric cases have reported specific variants of genes coding for receptors in serotonin, glutamate and dopamine pathways, as well as transcription and neurotrophic factors (13, 83).

## Environmental and Epigenetic Risk Factors

Because monozygotic twins (with identical DNA) show at most a 50% concordance for OCD (84), it is clear that epigenetic factors (modification of gene expression without change in DNA sequence) and non-genetic factors are equally or even more important. Indeed, more than half of all new cases of new onset OCD occur *without* a positive first-degree family history of OCD, so called “*sporadic*” cases (85). While sporadic cases may still have a genetic cause due to a new mutation (86), the frequent appearance of non-familial cases has led to interest in epigenetic triggers and non-shared environmental factors (85), especially as their occurrence cannot be ascribed to an affected relative. Three areas of investigation of possible environmental etiological influences which may be especially relevant to pediatric OCD include studies documenting higher rates of perinatal injury, acute onsets following infection with presumptive immune and/or inflammatory processes, and life events experienced as traumatic.

### Adverse Perinatal Risk Factors

Lensi et al. (87) reported that boys with OCD had elevated rates of adverse perinatal events, such as breech birth or low Apgar scores suggesting hypoxia. Geller et al. (88) compared perinatal history between 130 youths with OCD to 49 matched controls ascertained in a family genetic study and found maternal pregnancy histories of illness that needed medical attention, (*x*^2^ = 8.61, *p* = 0.003), and higher rates of labor difficulties such as induction, forceps, or prolonged labor (*x*^2^ = 7.51, *p* = 0.006). There was a positive correlation between these adverse labor events and earlier age at onset of OCD, greater symptom severity, and presence of concurrent disorders including chronic tics, anxiety, depression and ADHD. These early clinical observations were supported by a large epidemiological study of more than 2.4 million singleton births using the Swedish birth registry over a 24 year period that identified over 17,000 cases of OCD. After controlling for shared familial confounds, a number of adverse perinatal risk factors were associated with OCD including maternal smoking during pregnancy (HR, 1.20; 95% CI, 1.13–1.28), breech presentation (HR, 1.26; 95% CI, 1.15–1.39), and delivery by cesarean section (HR, 1.09; 95% CI, 1.04–1.15) (11). Additionally, low and high birth weight (1,501–2,500,g and >4,500,g, respectively) were related to a slightly higher risk for OCD (LBW: HR, 1.10; 95% CI, 1–1.21; HBW: HR, 1.17; 95% CI, 1.07–1.27) (11). Such epidemiological approaches provide a powerful method for finding epigenetic triggers with very small effect sizes and, although non-specific, indicate that OCD could have antecedents long before the disorder appears, and during periods of vulnerable neural maturation. These findings are consistent with those of Vasconcelos et al. (89), whose research similarly showed a relationship between clinical expression of OCD and perinatal complications in adult cases of OCD compared to controls, including cesarean delivery (*p* = 0.005), prolonged labor (*p* < 0.001), and nuchal cord entanglement (*p* = 0.05), as well as other postnatal complications. In one study that examined perinatal complications among individuals with chronic tic disorders (age range 3–79 years), the authors found that pregnancy, delivery, and postnatal complications were associated with comorbid OCD (12).

### Psychosocial Stress

Ironically, data that shows an association between traumatic life events and OCD affecting youth is extremely sparse, perhaps because definitive linkage is hard to establish. In contrast, the association between OCD and PTSD has been reported in numerous studies of adults with OCD (90) either with sequential or concurrent onsets, including in military veterans (91, 92). In one of the only pediatric studies to report on this potential association, Lafleur et al. (93) examined a cohort of 263 pediatric cases of OCD, finding child and parental reports of salient traumatic and stressful life events at higher rates than matched controls. Domestic violence, sexual or physical assault and forced home entry were some examples reported by youth and families and in some cases, the thematic content of obsessional fears and rituals mirrored the nature of the trauma (e.g., checking for safety repeatedly following a serious domestic assault on a parent) (93). It may be difficult to determine whether any given event reaches a threshold considered as “*trauma*” and further to demonstrate statistical significance across pediatric cohorts but for a subset of children, OCD will sometimes follow severe psychological stress. One interesting study illuminates how trauma may be translated into fear related behaviors at the molecular level. McGregor et al. (94) examined children exposed to trauma using the childhood trauma questionnaire (CTQ) and several polymorphisms in genes encoding mono-amine oxidase A and B (MAO-A, MAO-B) and catechol-O-methyl transferase (COMT). Gene by environment interactions suggested that these haplotypes “*interacted*” with childhood sexual trauma to increase risk for OCD in youth, providing a potential epigenetic mechanism of action for adverse psychosocial experiences (95).

### Immunity, Infection, and Inflammation

In 1997, Swedo et al. (96) described a group of children who developed OCD subsequent to infection with group A beta-hemolytic streptococcal (GABHS or strep) infection and introduced the hypothesis of pediatric autoimmune neuropsychiatric disorder associated with streptococcus (PANDAS). This hypothesis posits that an immune response to streptococcal infection may be a causative antecedent of OCD in some youth. Evidence to support such a hypothesis derives from observations of other neurobehavioral sequelae of group A strep, notably Sydenham's chorea, implicating basal ganglia dysfunction (97). Some level of confirmation for the etiological role of streptococcal infection was also derived from the OCD Genetics Association Collaborative (genome-wide association) Study (OCGAS GWAS), where high rates of infection were seen in pediatric OCD cases. Putative immune factors, for example, cross reactive anti-strep antibodies affecting circuits implicated in OCD and causing inflammation and dysfunction, are thought to cause a range of neurobehavioral symptoms including, but not limited to, OCD. Although over two decades have passed, the academic discussion about the validity of such an etiology remains highly controversial, because such antibodies have yet to be reliably demonstrated and other biomarkers have not been consistently identified. Diagnostic criteria include (1) OCD and/or a tic disorder; (2) prepubertal onset between 3 and 12 years of age, or Tanner stage I or II; (3) episodic course (abrupt onset and/or exacerbations); (4) symptom onset/exacerbation temporally linked to *documented* GABHS infections on two occasions; (5) association with neurological abnormalities (96). These criteria do not operationalize several important elements including the temporal duration between GABHS and onset, the data needed to definitively *document* GABHS, or the nature of neurological abnormalities (usually considered to be chorea or chorea-like movements or a loss of fine motor skills). The relevance for an pediatric subtype derives from the fact that nearly all youth are exposed to GABHS by early adolescence and develop antibodies. Therefore, new GABHS infections are far less common after puberty due to the heard immunity of the adolescent and their peers.

For all supporting studies there appear to be studies with conflicting findings. For example, Mell et al. (98) found an association between OCD/Tourette's disorder and GABHS while others have refuted this finding (99, 100). Giedd et al. (101) described acute and transient structural abnormalities in the brains of some children with putative PANDAS but such findings have not been reproduced. Some suggest that transient increases in tics and OCD are well-known sequelae of many infections and other physiological stressors and not unique to GABHS (102). The search for specific culpable antibodies that co-localize to brain targets of interest has generally been unsuccessful. While anti-streptococcal antibodies can easily be measured in serum (103), presence of such antibodies or indeed other anti-neuronal antibodies, has not been consistently demonstrated. Two very recent studies demonstrated binding of antibodies from sera of children with putative PANDAS to cholinergic interneurons in the striatum with a subsequent alteration in their function, which represents the first such definitive finding but requires replication (104).

One adult study (105) showed inflammation in nuclei of the basal ganglia in adults with OCD compared with controls, but neuroimaging data in putative PANDAS youth has been sparse. Biomarker studies in other psychiatric disorders have frequently been reported to show evidence of inflammation (106), so that this may not be unique to OCD. Indeed Fullana et al. (107) reviewed this literature and found none to be sufficiently sensitive or specific for OCD. Humoral immunodeficiency has also been linked to OCD onset in children (97) as well as psychiatric disorders and suicide in adults (108) perhaps suggesting increased risk for infections and subsequent pathogenic immune responses.

Treatment studies represent another approach to validate the immune-mediated etiology using anti-microbials (109, 110), non-steroidal anti-inflammatory agents such as naproxen sodium (111) and cyclo-oxygenase inhibitors such as celecoxib (112, 113), but these studies involved several psychiatric disorders, suggesting a non-specific effect. However, one study reported an improvement in OCD symptoms specifically (114). Finally, direct immune modulation using intravenous immunoglobulin failed to demonstrate a benefit in a randomized placebo-controlled trial in OCD-affected PANDAS youth although several methodological limitations leave open the question of whether this finding was true or simply a failed trial (115). Similarly, a recent prospective study failed to show exacerbation of tics following documented streptococcal infections (116).

While the clinical studies have provided, at best, contradictory evidence, more convincing evidence comes from epidemiological studies that by definition, are retrospective and agnostic to any pre-existing notions of the validity of immune-mediated neuropsychiatric illness. Mataix-Cols et al. (117) examined the records of over seven million youth from the Swedish birth registry born between 1940 and 2007 (mostly before PANDAS had been described) and showed a significantly higher rate of autoimmune illnesses in families of those youth affected by OCD and Tourette's disorder. Of course, correlation does not equal causation, and both may share underlying etio-pathological mechanisms, but the link between OCD and immune illness appears well-established and provides an important avenue for future research. Orlovska et al. (118) also analyzed the medical records of more than one million youth between birth and age 17 years from the Danish birth registry and found that all psychiatric disorders were over-represented in those with a history of both streptococcal and non-streptococcal. Of interest, tic and OCD showed the greatest increased risk among disorders studied (119).

In 2010, a scientific “white paper” consensus group at the NIMH child psychiatry branch suggested decoupling the acute onset of neuropsychiatric disorders in children from specific pathogens (GABHS) and expanded the clinical presentations to include avoidant/restrictive food intake disorder (ARFID) (120). Disordered eating behaviors have been described in a population-based prospective cohort study of over half a million young women identified thought the Danish longitudinal health register over a 6-year period (121). Anorexia nervosa, bulimia nervosa and eating disorder not otherwise specified was significantly more prevalent among females previously hospitalized for severe infections as well as those who had received anti-microbial treatment. This expanded constellation of clinical presentations was coined pediatric acute onset neuropsychiatric disorders or PANS. There are advantages to this approach (opening up research to other possible pathogens and pathogenetic mechanisms and perhaps expanding treatment options for a subset of affected youth), as well as disadvantages (linkage between infection and subsequent is inferred from acuity of onset and proximity to an infection but with no specificity). If PANDAS and PANS are conceived as variants of an autoimmune encephalitis, then the anti-N-methyl-d-aspartate (NMDA) receptor antibody mediated neurological disorder (122) may serve as a model. However, in PANS and PANDAS, the presumptive immune trigger is an exogenous infection of some kind, and it is the immune response that causes inflammatory-mediated neurobehavioral change, either through innate or adaptive immune responses, including cross reactivity of antibodies and cytokine activation.

In summary, evidence is accumulating incrementally that a subset of cases of pediatric OCD are triggered by infections and mediated by immune and inflammatory processes (123).

## Neuroimaging findings

Structural and functioning imaging research over several decades have shown great concordance across studies regarding the underlying neurocircuitry of OCD (53). OCD is associated with abnormal findings in cortico-striato-thalamo-cortical (CSTC) circuitry which originate in the prefrontal cortex connect to the striatum, pallidum and thalamus and then loop back to cortical areas (124). Cortical areas consistently implicated using structural MRI include the orbitofrontal cortex (OFC), anterior cingulate cortex (ACC) and striatum (125) as well as thalamus (126). A finer grained understanding of the involvement of these regions is provided by fMRI studies that have identified specific roles of the dorsal ACC (dACC), medial and lateral OFC, and connections between amygdala and cortex (127, 128).

While a recent imaging study of youth with subclinical OC symptoms showed no morphological abnormalities at all, many reviews (129) of the neurocircuitry in adults and children with OCD show concordant abnormal findings (130–132). Confirmation derives from the recent ENIGMA Consortium meta-analysis of 16 pediatric and 30 adult OCD datasets that found cortical thinning in parietal regions in both age groups. However, some differences between age groups were also noted. The ENIGMA study reported asymmetries in thalamus and pallidum volumes in children that were not seen in the adult studies (133, 134). A recent structural MRI study of 2,551 youth enrolled in the Generation R study (135) showed a significant reduction in total brain volume in probable OCD cases compared to non-OCD healthy controls, and a significant increase in thalamic volume (126). While the same cortical thinning that was seen in ENIGMA was not demonstrated in this study, the mean age at imaging was much younger in the R Generation study so that developmental changes may account for discrepancies. Along with earlier findings by Gilbert et al. (136) cumulative evidence has led to a focus on the thalamus as an area that may distinguish pediatric and adult OCD (126). In contrast, an fMRI study (137) reported involvement of the temporal poles during symptom provocation in children with OCD compared to matched healthy controls, rather than the CSTC loops generally reported. It is important to emphasize that morphology and circuitry will mature considerably throughout childhood and adolescence and at differing and variable rates (138) with implications for both imaging research as well as future non-invasive neuromodulation treatment protocols.

## Neuropsychological findings

Although many studies have examined neuropsychological test performance in OCD subjects, in both adults and in affected youth, the literature is rather inconsistent. Salient domains include attention, executive function, short-term memory and visuospatial function. Academic difficulties, seen frequently in youth with OCD, could simply reflect the intrusive effect of primary obsessions and high anxiety, or some deficits related to abnormalities in the CSTC not due to OCD at all (139). Efforts to identify significant performance deficits in neurocognitive function in youth and throughout development may yield findings relevant to translational investigations in OCD.

In one study of 102 youth with OCD and matched controls, a standard battery of tests showed reductions in processing speed and timed visuospatial test performance (139, 140). Working memory evaluation showed a similar pattern with deficits in timed tests. Processing speed weaknesses may therefore be central to deficits in neuropsychological performance in youth. Notably, these were *relative* weaknesses, inasmuch as the overall scores remained within the “normal” range (5^th^-95^th^%) but these may have ecologically important consequences in academic settings (140). Similarly, in adults, deficits in non-verbal memory, planning, processing speed and inhibition has also been reported consistently (141).

Interest in the heritability of these deficits has been explored in some familial studies of neurocognitive performance, and have extended to cognitive flexibility and set shifting, decision making and visuospatial integration (142, 143). In a recent pediatric study of OCD youth and their first-degree unaffected relatives designed to examine neuropsychological endophenotypes, poorer proactive control and initial concept formation, seen in tests of set shifting and inhibitory control were found to be heritable (6).

## Treatment response

The very high prevalence of comorbid psychiatric disorders associated with OCD in youth, in clinical and also non-referred epidemiological cohorts, present real challenges in treatment which are not seen in adult cases. For example, high rates of Tourette's syndrome and chronic tic disorders as well as ADHD (144, 145) mean that a child's OCD cannot be treated in isolation (146). While CBT is the first recommended intervention for all affected youth, it is notable that in the management of OCD, selective serotonin re-uptake inhibitors (SSRIs) (147) are considered first-line medication treatments. In contrast, ADHD responds best to stimulant medication approaches while tics are most often treated with either alpha agonist medications or dopamine blockers. In other words, pharmacotherapy approaches for each comorbid condition diverge markedly despite the frequent triad of these conditions. Add to this the increased risk of behavioral activation and suicidal ideation accompanying SSRIs in youth (148), the potential for increased anxiety, obsessions and tics with use of stimulants and the risk for adverse mood effects with alpha agonists, and the pharmacological approach in affected youth may require increased complexity compared to adult OCD cases. While CBT is clearly the treatment of choice for youth with OCD (147, 149, 150), more severe illness and concurrent psychopathology are indications for consideration of introduction of medication. Poor insight and low levels of family cohesion may also impede delivery of successful CBT. Youth with OCD who represent putative post-infectious, immune-mediated and/or inflammatory etiology have received a variety of anti-microbial and immune modulating treatments (109, 110, 115, 151) but none have yet shown consistent efficacy that permits recommendation for routine use.

## Course and Outcome

Again, comorbid externalizing symptoms have been reported to affect quality of life ratings at baseline and also with treatment (152). Many researchers have suggested that poorer treatment outcomes may be due to greater levels of family accommodation (FA) and this is especially relevant to pediatric cases (69, 71, 73, 74). As well, concurrent psychopathology has been shown to reduce response rates, particularly for conditions prevalent in pediatric cases. For example, comorbid tic disorders and ADHD reduced response rates to 53 and 59%, respectively, in a randomized controlled trial of paroxetine (153), and relapse was also higher in comorbid cases. March et al. (154) found the same poor response in youth with OCD who had a comorbid tic disorder, again suggesting that this pediatric subtype may be distinct in important ways.

Remission rates of OCD in youth treated with CBT are fair with partial remission reported in 53% and full remission in 27%, but also with some risk of relapse (155, 156). Outcome in youth appear to be better than in adults with some children becoming subclinical or remitting entirely over time (10, 150), whereas only 16.9% of adults were shown to achieve full remission in a 5 year longitudinal study by Eisen et al. (157). The Nordic Long Term OCD Treatment Study (NordLOTS) that used a stepped treatment protocol showed 90% response and 73% rate of clinical remission at 3 year follow-up (150). Fatori et al. (158) found that, treatment sequence with either SSRI or CBT did not affect outcome when the second treatment arm was added as needed.

## Summary

In this review, we have detailed the many differences between pediatric or pediatric OCD and OCD that onsets in adults. These numerous distinctions are summarized in Table 2. Distinct age peaks lend credence to the notion of differing pathophysiological mechanisms rather than simply increased genetic loading leading to earlier onsets. Familial patterns, comorbid disorders, phenotypic presentations, etiologies, neurocognitive findings, treatment and outcome are also different, and the many developmental factors that distinguish pediatric cases have been elucidated. Keeping in mind that development throughout the pediatric years is rapid and that accompanying neuronal maturation occurs with similar rapid synchrony, these factors consequently may greatly affect the presentation and research findings in affected youth and adults. Therefore, while there is substantial evidence to support the notion of a “developmental” pediatric subtype of OCD, clarification must await further translational and genetic studies.

**Table 2:** Comparison of pediatric and adult-onset OCD.

**Areas of Investigation**	**Pediatric OCD**	**Adult-onset OCD**
Prevalence	0.84% prevalence (1/3–1/2 remission rate)	1–3% prevalence
Age at onset	9–10 (with an SD of ±2.5 years)	22–24 years
Gender ratio	F > M	F > M
OCD symptoms	*Children-* Intrusive fears of harm or loss of attachment figures. Hoarding. Symmetry and ‘just right' phenomena. Fewer concrete cognitive obsessions. *Adolescents-* sexual, moral and religious themes, scrupulosity. Contamination fears.	Contamination, more stable over time and across fewer categories of obsessions/compulsion types.
Insight	Limited- only 63% have good or excellent insight	13.8–30.7% have poor to no insight
Comorbidity	Up to 80%- Mood and anxiety conditions, ADHD, Tic disorders, ODD, DMDD, ASD (~5%)	Mood and anxiety disorders
Family role	Greater family involvement leads to worse OCD symptoms and greater functional impairment	Family accommodation also seen in relatives of adult onset OCD but less direct involvement in rituals
Genetics	26% risk of OCD in a first degree relative	12% risk of OCD in a first degree relative
Adverse perinatal risk factors	Increased rates, especially in boys with OCD	Associated with an earlier age of OCD onset
Psychosocial stress	Increased rate of traumatic and stressful life events	Association with PTSD
Immune and inflammatory factors	Possible association with GABHS infections. Link with humeral immunodeficiency	Possible basal ganglia inflammation. Link with humeral immunodeficiency
Neurocircuitry	Similar to adult findings, possible increased assymetry of thalamus and palladium volumes and increase in total brain volume	CSTC: OFC, ACC, striatum, thalamus
Neuropsychological findings	Deficits in working memory, visuospatial test performance and processing speed.	Inconsistent- salient domains include attention, executive function, short-term memory and visuospatial function
Treatment Response	Complicated by prevalence and diversity of co-morbidities, and increased risk of behavioral activation and suicidal ideation accompanying SSRIs in youth	SSRIs and CBT
Course and Outcome	Worse outcomes with co-morbid externalizing conditions and greater degrees of family accommodation. Overall higher rates of remission and symptoms becoming subclinical	Few cases of full remission over time

## Author Contributions

DG, GJ, and SH declare that they have contributed substantially to the content and production of this review and agree to be accountable for the content of this work. All authors contributed to the article and approved the submitted version.

## Conflict of Interest

DG has received grant or research support from the Eunice Kennedy Shriver National Institute of Child Health and Human Development subcontract with Duke Clinical Research Center Pediatric Trials Network, the National Institute of Mental Health, Biohaven Pharmaceuticals, Boehringer Ingelheim, Eli Lilly and Co., Forest Pharmaceuticals, GlaxoSmithKline, the International OCD Foundation, Neurocrine Biosciences, Nuvelution Pharma, Peace of Mind Foundation, Pfizer, Solvay, Syneos Health, Teva Pharmaceutical Industries, Emalex, the OCD Foundation, and the Tourette Association of America.He has served as a consultant to the Arlington Youth Counseling Center. He has served on the editorial board of the Journal of the American Academy of Child and Adolescent Psychiatry, Comprehensive Psychiatry and Annals of Clinical Psychiatry. He has received honoraria from the Massachusetts Psychiatry Academy and the American Academy of Child and Adolescent Psychiatry. He has previously held stock options/ownership in Assurex Health, Revolution Clinics and CD Services of America. The remaining authors declare that the research was conducted in the absence of any commercial or financial relationships that could be construed as a potential conflict of interest.

## References

[1] Association AP. Diagnostic and Statistical Manual of Mental Disorders. 5th ed. DSM-5. Arlington, TX: American Psychiatric Publishing (2013).

[2] Geller DA Biederman J Stewart SE Mullin B Farrell C Wagner KD. Impact of comorbidity on treatment response to paroxetine in pediatric obsessive-compulsive disorder: is the use of exclusion criteria empirically supported in randomized clinical trials? J Child Adolesc Psychopharmacol. (2003) 13:19–29. 10.1089/104454603322126313 12880497

[3] Stewart ES Rosario MC Brown TA Carter AS Leckman JF Sukhodolsky DG. Principal components anaylsis of obsessive-compulsive disorder symptoms in children and adolescents. Biol Psychiatry. (2007) 61:285–91. 10.1016/j.biopsych.2006.08.040 17161383

[4] Brezinka V Mailander V Walitza S. Obsessive compulsive disorder in very young children—a case series from a specialized outpatient clinic. BMC Psychiatry. (2020) 20:366. 10.1186/s12888-020-02780-0 32653035 PMC7353707

[5] Geller DA McGuire JF Orr SP Pine DS Britton JC Small BJ. Fear conditioning and extinction in pediatric obsessive compulsive disorder. Ann Clin Psychiatry. (2017) 29:17–26. 28207912 PMC5964984

[6] Abramovitch A De Nadai AS Geller DA. Neurocognitive endophenotypes in pediatric OCD probands, their unaffected parents and siblings. Prog Neuropsychopharmacol Biol Psychiatry. (2021) 110:110283. 10.1016/j.pnpbp.2021.110283 33609605 PMC8222154

[7] Rosario-Campos MC Leckman JF Mercadante MT Shavitt RG Prado HS Sada P. Adults with early-onset obsessive-compulsive disorder. Am J Psychiatry. (2001) 158:1899–903. 10.1176/appi.ajp.158.11.1899 11691698

[8] Taylor S. Early versus late onset obsessive-compulsive disorder: evidence for distinct subtypes. Clin Psychol Rev. (2011) 31:1083–100. 10.1016/j.cpr.2011.06.007 21820387

[9] Walitza S Wendland JR Gruenblatt E Warnke A Sontag TA Tucha O. Genetics of early-onset obsessive-compulsive disorder. Eur Child Adolesc Psychiatry. (2010) 19:227–35. 10.1007/s00787-010-0087-7 20213231

[10] Stewart SE Geller DA Jenike M Pauls D Shaw D Mullin B. Long term outcome of pediatric obsessive compulsive disorder: a meta-analysis and qualitative review of the literature. Acta Psychiatrica Scandinavica. (2004) 110:4–13. 10.1111/j.1600-0447.2004.00302.x 15180774

[11] Brander G Rydell M Kuja-Halkola R Fernandez de la Cruz L Lichtenstein P Serlachius E. Association of perinatal risk factors with obsessive-compulsive disorder: a population-based birth cohort, sibling control study. JAMA Psychiatry. (2016) 73:1135–44. 10.1001/jamapsychiatry.2016.2095 27706475

[12] Abdulkadir M Tischfield JA King RA Fernandez TV Brown LW Cheon KA. Pre- and perinatal complications in relation to Tourette syndrome and co-occurring obsessive-compulsive disorder and attention-deficit/hyperactivity disorder. J Psychiatr Res. (2016) 82:126–35. 10.1016/j.jpsychires.2016.07.017 27494079 PMC5026935

[13] Grunblatt E Marinova Z Roth A Gardini E Ball J Geissler J. Combining genetic and epigenetic parameters of the serotonin transporter gene in obsessive-compulsive disorder. J Psychiatr Res. (2018) 96:209–17. 10.1016/j.jpsychires.2017.10.010 29102815

[14] Fineberg N Hollander E Pallanti S Walitza S Grunblatt E Dell'Osso B. Clinical advances in obsessive compulsive disorder: a position statement by the international college of obsessive compulsive spectrum disorders. Inter Clin Psychopharmacology. (2020) 35:173–93. 10.1097/YIC.0000000000000314 32433254 PMC7255490

[15] Rapoport J. The boy who couldn't stop washing: the experience and treatment of obsessive-compulsive disorder. Am J Psychiatry. (1991) 148:678. 10.1176/ajp.148.5.678-a

[16] Flament M Whitaker A Rapoport J Davies M Berg C Kalikow K. Obsessive compulsive disorder in adolescence: an epidemiological study. J Am Acad Child Adolesc Psychiatry. (1988) 27:764–71. 10.1097/00004583-198811000-00018 3264280

[17] Heyman I Fombonne E Simmons H Ford T Meltzer H Goodman R. Prevalence of obsessive–compulsive disorder in the British nationwide survey of child mental health. British J Psych. (2001) 179:324–9. 10.1192/bjp.179.4.324 11581112

[18] Browne HA Hansen SN Buxbaum JD Gair SL Nissen JB Nikolajsen KH. Familial clustering of tic disorders and obsessive-compulsive disorder. JAMA Psychiatry. (2015) 72:359–66. 10.1001/jamapsychiatry.2014.2656 25692669

[19] Ruscio AM Stein DJ Chiu WT Kessler RC. The epidemiology of obsessive-compulsive disorder in the National Comorbidity Survey Replication. Mol Psychiatry. (2010) 15:53–63. 10.1038/mp.2008.94 18725912 PMC2797569

[20] Weissman M Bland R Canino G Greenwald S Hwu H Lee C. The cross national epidemiology of obsessive compulsive disorder. J Clin Psychiatry. (1994) 55:5–10. 8077177

[21] Dell'Osso B Benatti B Hollander E Fineberg N Stein DJ Lochner C. Childhood, adolescent and adult age at onset and related clinical correlates in obsessive-compulsive disorder: a report from the International College of Obsessive-Compulsive Spectrum Disorders (ICOCS). Int J Psychiatry Clin Pract. (2016) 20:210–7. 10.1080/13651501.2016.1207087 27433835

[22] Geller D Biederman J Faraone SV Frazier J Coffey BJ Kim GS. Clinical correlates of obsessive compulsive disorder in children and adolescents referred to specialized and non-specialized clinical settings. Depress Anxiety. (2000) 11:163–8. 10945136 10.1002/1520-6394(2000)11:4<163::AID-DA3>3.0.CO;2-3

[23] Rasmussen SA Eisen J. The epidemiology and clinical features of obsessive compulsive disorder. Psychiatr Clin. (1992) 15:743–58. 10.1016/S0193-953X(18)30205-3 1461792

[24] Pauls D Alsobrook II J Goodman W Rasmussen S Leckman J. A family study of obsessive-compulsive disorder. Am J Psychiatry. (1995) 152:76–84. 10.1176/ajp.152.1.76 7802125

[25] Geller D Biederman J Faraone SV Bellorde CA Kim GS Hagermoser LM. Disentangling chronological age from age of onset in children and adolescents with obsessive compulsive disorder. Int J Neuropsychopharmacol. (2001) 4:169–78. 10.1017/S1461145701002395 11466167

[26] Tao Z Shi A Zhao J. Epidemiological perspectives of diabetes. Cell Biochem Biophys. (2015) 73:181–5. 10.1007/s12013-015-0598-4 25711186

[27] Reinehr T. Type 2 diabetes mellitus in children and adolescents. World J Diabetes. (2013) 4:270–81. 10.4239/wjd.v4.i6.270 24379917 PMC3874486

[28] Fineberg NA Brown A Reghunandanan S Pampaloni I. Evidence-based pharmacotherapy of obsessive-compulsive disorder. Int J Neuropsychopharmacol. (2012) 15:1173–91. 10.1017/S1461145711001829 22226028

[29] Dalsgaard S Thorsteinsson E Trabjerg BB Schullehner J Plana-Ripoll O Brikell I. Incidence rates and cumulative incidences of the full spectrum of diagnosed mental disorders in childhood and adolescence. JAMA Psychiatry. (2019) 77:155–64. 10.1001/jamapsychiatry.2019.3523 31746968 PMC6902162

[30] Mathis M Alvarenga P Funaro G Torresan R Moraes I Torres A. Gender differences in obsessive-compulsive disorder- a literature review. Rev Bras Psiquiatr. (2011) 33:390–9. 10.1590/S1516-44462011000400014 22189930

[31] Millet N Longworth J Arcelus J. Prevalence of anxiety symptoms and disorders in the transgender population: a systematic review of the literature. Int J Transgenderism. (2016) 18:27–38. 10.1080/15532739.2016.1258353

[32] Leonard HL Goldberger EL Rapoport JL Cheslow DL Swedo SE. Childhood rituals: normal development or obsessive-compulsive symptoms. J Am Acad Child Adolesc Psychiatry. (1990) 29:17–23. 10.1097/00004583-199001000-00004 2295573

[33] Storch EA Wu MS Small BJ Crawford EA Lewin AB Horng B. Mediators and moderators of functional impairment in adults with obsessive-compulsive disorder. Compr Psychiatry. (2014) 55:489–96. 10.1016/j.comppsych.2013.10.014 24342055

[34] Storch EA Caporino NE Morgan JR Lewin AB Rojas A Brauer L. Preliminary investigation of web-camera delivered cognitive-behavioral therapy for youth with obsessive-compulsive disorder. Psychiatry Res. (2011) 189:407–12. 10.1016/j.psychres.2011.05.047 21684018

[35] Hojgaard D Skarphedinsson G Ivarsson T Weidle B Nissen JB Hybel KA. Hoarding in children and adolescents with obsessive-compulsive disorder: prevalence, clinical correlates, and cognitive behavioral therapy outcome. Eur Child Adolesc Psychiatry. (2019) 28:1097–106. 10.1007/s00787-019-01276-x 30656432

[36] Labad J Menchon JM Alonso P Segalas C Jimenez S Jaurrieta N. Gender differences in obsessive-compulsive symptom dimensions. Depress Anxiety. (2008) 25:832–8. 10.1002/da.20332 17436312

[37] Geller D Biederman J Agranat A Cradock K Hagermoser LM Kim GS. Developmental aspects of obsessive compulsive disorder: Findings in children, adolescents and adults. J Nerv Ment Dis. (2001) 189:471–7. 10.1097/00005053-200107000-00009 11504325

[38] Rettew DC Swedo SE Leonard HL Lenane MC Rapoport JL. Obsessions and compulsions across time in 79 children and adolescents with obsessive-compulsive disorder. J Am Acad Child Adolesc Psychiatry. (1992) 31:1050–6. 10.1097/00004583-199211000-00009 1429404

[39] Goodman WK Rasmussen SA Price LH. Children's Yale-Brown Obsessive Compulsive Scale (CY-BOCS). 1st ed. New Haven, CT: Yale University (1986).

[40] Cervin M Miguel EC Guler AS Ferrao YA Erdogdu AB Lazaro L. Towards a definitive symptom structure of obsessive-compulsive disorder: a factor and network analysis of 87 distinct symptoms in 1366 individuals. Psychol Med. (2021) 1–13. 10.1017/S0033291720005437 33557980 PMC9693708

[41] Storch E Jones AM Lack CW Ale C Sulkowski M Lewin A. Rage attacks in pediatric obsessive-compulsive disorder- phenomenology and clinical correlates. J Am Acad Child Adolesc Psychiatry. (2012) 51:582–92. 10.1016/j.jaac.2012.02.016 22632618

[42] Guzick AG Geller DA Small BJ Murphy TK Wilhelm S Storch EA. Irritability in children and adolescents with OCD. Behav Ther. 51:582–92. (2020). 10.1016/j.beth.2020.11.001 PMC8217718 34134828

[43] Brandes CM Herzhoff K Smack A Tackett JL. The p factor and the n factor- associations between the general factors of psychopathology and neuroticism in children. Clin Psychol Sci. (2019) 7:1266–84. 10.1177/2167702619859332

[44] Copeland WE Angold A Costello E Egger H. Prevalence, comorbidity, and correlates of DSM-5 proposed disruptive mood dysregulation disorder. Am J Psychiatry. (2013) 170:173–79. 10.1176/appi.ajp.2012.12010132 23377638 PMC3573525

[45] Evans SC Bonadio FT Bearman SK Ugueto AM Chorpita BF Weisz JR. Assessing the irritable and defiant dimensions of youth oppositional behavior using CBCL and YSR items. J Clin Child Adolesc Psychol. (2020) 49:804–19. 10.1080/15374416.2019.1622119 31276433

[46] Shimshoni Y Lebowitz ER Brotman MA Pine DS Leibenluft E Silverman WK. Anxious-irritable children: a distinct subtype of childhood anxiety? Behav Ther. (2020) 51:211–22. 10.1016/j.beth.2019.06.005 32138933 PMC7080292

[47] Sukhodolsky DG Smith SD McCauley SA Ibrahim K Piasecka JB. Behavioral interventions for anger, irritability, and aggression in children and adolescents. J Child Adolesc Psychopharmacol. (2016) 26:58–64. 10.1089/cap.2015.0120 26745682 PMC4808268

[48] Kircanski K Craske MG Averbeck BB Pine DS Leibenluft E Brotman MA. Exposure therapy for pediatric irritability: theory and potential mechanisms. Behav Res Ther. (2019) 118:141–9. 10.1016/j.brat.2019.04.007 31085355 PMC6590706

[49] Selles RR Hojgaard D Ivarsson T Thomsen PH McBride NM Storch EA. Avoidance, insight, impairment recognition concordance, and cognitive-behavioral therapy outcomes in pediatric obsessive-compulsive disorder. J Am Acad Child Adolesc Psychiatry. (2020) 59:650–9. e2. 10.1016/j.jaac.2019.05.030 31228561 PMC7179819

[50] Jacob ML Larson MJ Storch EA. Insight in adults with obsessive-compulsive disorder. Compr Psychiatry. (2014) 55:896–903. 10.1016/j.comppsych.2013.12.016 24445116

[51] Peris TS Rozenman MS Sugar CA McCracken JT Piacentini J. Targeted family intervention for complex cases of pediatric obsessive-compulsive disorder: a randomized controlled trial. J Am Acad Child Adolesc Psychiatry. (2017) 56:1034–42. e1. 10.1016/j.jaac.2017.10.008 29173737 PMC5875916

[52] Kessler R Ormel J Petukhova M McLaughlin K Green J Russo L. Development of lifetime comorbidity in the world health organization world mental health surveys. JAMA. (2011) 68:90–100. 10.1001/archgenpsychiatry.2010.180 21199968 PMC3057480

[53] Chamberlain SR Blackwell AD Fineberg NA Robbins TW Sahakian BJ. The neuropsychology of obsessive compulsive disorder: the importance of failures in cognitive and behavioural inhibition as candidate endophenotypic markers. Neurosci Biobehav Rev. (2005) 29:399–419. 10.1016/j.neubiorev.2004.11.006 15820546

[54] World Health Organization. ICD-10 Classification of Mental and Behavioural Disorders. Geneva: World Health Organization (1992).

[55] Volkmar F Siegel M Woodbury-Smith M King B McCracken J State M. Practice parameter for the assessment and treatment of children and adolescents with autism spectrum disorder. J Am Acad Child Adolesc Psychiatry. (2014) 53:237–57. 10.1016/j.jaac.2013.10.013 24472258

[56] Leckman JF Pauls DL Zhang H Rosario-Campos MC Kastovich L Kidd KK. Obsessive-compulsive symptom dimensions in affected sibling pairs diagnosed with gilles de la tourette syndrome. Am J Med Genet B Neuropsychiatr Genet. (2003) 116B:60–8. 10.1002/ajmg.b.10001 12497616

[57] Storch EA Merlo LJ Larson MJ Geffken GR Lehmkuhl HD Jacob ML. Impact of comorbidity on cognitive-behavioral therapy response in pediatric obsessive-compulsive disorder. J Am Acad Child Adolesc Psychiatry. (2008) 47:583–92. 10.1097/CHI.0b013e31816774b1 18356759

[58] Geller DA Wagner KD Emslie G Murphy T Carpenter DJ Wetherhold E. Paroxetine treatment in children and adolescents with obsessive-compulsive disorder: a randomized, multicenter, double-blind, placebo-controlled trial. J Am Acad Child Adolesc Psychiatry. (2004) 43:1387–96. 10.1097/01.chi.0000138356.29099.f1 15502598

[59] Calvocoressi L Lewis B Harris M Trufan S Goodman W McDougle C. Family accommodation in obsessive-compulsive disorder. Am J Psychiatry. (1995) 152:441–3. 10.1037/t29858-000 7864273

[60] Lebowitz ER Panza KE Su J Bloch MH. Family accommodation in obsessive-compulsive disorder. Expert Rev Neurother. (2012) 12:229–38. 10.1586/ern.11.200 22288678 PMC4011018

[61] Peris TS Bergman RL Langley A Chang S McCracken JT Piacentini J. Correlates of accommodation of pediatric obsessive-compulsive disorder: parent, child, and family characteristics. J Am Acad Child Adolesc Psychiatry. (2008) 47:1173–81. 10.1097/CHI.0b013e3181825a91 18724255 PMC3378323

[62] Boeding SE Paprocki CM Baucom DH Abramowitz JS Wheaton MG Fabricant LE. Let me check that for you: symptom accommodation in romantic partners of adults with obsessive-compulsive disorder. Behav Res Ther. (2013) 51:316–22. 10.1016/j.brat.2013.03.002 23567474

[63] Wu MS Lewin AB Murphy TK Geffken GR Storch EA. Phenomenological considerations of family accommodation: Related clinical characteristics and family factors in pediatric obsessive–compulsive disorder. J Obsess Compuls Relat Dis. (2014) 3:228–35. 10.1016/j.jocrd.2014.05.003

[64] Nestadt G Samuels J Riddle M Bienvenu J Liang K-Y LaBuda M. A family study of obsessive-compulsive disorder. Arch Gen Psychiatry. (2000) 57:358–63. 10.1001/archpsyc.57.4.358 10768697

[65] Jacoby RJ Heatherington L. Growing up with an anxious sibling: psychosocial correlates and predictors of sibling relationship quality. Curr Psychol. (2015) 35:57–68. 10.1007/s12144-015-9360-8

[66] Calvocoressi L Mazure C Kasl S Skolnick J Fisk D Vegso S. Family accommodation of obsessive-compulsive symptoms: instrument development and assessment of family behavior. J Nerv Ment Dis. (1999) 187:636–42. 10.1097/00005053-199910000-00008 10535658

[67] Freeman J Sapyta J Garcia A Compton S Khanna M Flessner C. Family-based treatment of early childhood obsessive-compulsive disorder: the pediatric obsessive-compulsive disorder treatment study for young children (POTS Jr)—a randomized clinical trial. JAMA Psychiatry. (2014) 71:689–98. 10.1001/jamapsychiatry.2014.170 24759852 PMC4511269

[68] Jacoby RJ Smilansky H Shin JG Wu M Small BJ Wilhelm S. Predictors of change in family accommodation during exposure therapy for pediatric OCD. Partnering with families in therapy: leveraging family processes in the treatment of anxiety disorders. Paper presented at the 53^*rd*^ annual convention of the Association for Behavioral and Cognitive Therapies. Atlana, GA. (2019).

[69] Merlo LJ Lehmkuhl HD Geffken GR Storch EA. Decreased family accommodation associated with improved therapy outcome in pediatric obsessive-compulsive disorder. J Consult Clin Psychol. (2009) 77:355–60. 10.1037/a0012652 19309195 PMC2886196

[70] Albert U Baffa A Maina G. Family accommodation in adult obsessive-compulsive disorder: clinical perspectives. Psychol Res Behav Manag. (2017) 10:293–304. 10.2147/PRBM.S124359 29033617 PMC5614765

[71] Storch EA Geffken GR Merlo LJ Jacob ML Murphy TK Goodman WK. Family accommodation in pediatric obsessive-compulsive disorder. J Clin Child Adolesc Psychol. (2007) 36:207–16. 10.1080/15374410701277929 17484693

[72] Morgan J Caporino NE De Nadai AS Truax T Lewin AB Jung L. Preliminary predictors of within-session adherence to exposure and response prevention in pediatric obsessive–compulsive disorder. Child Youth Care Forum. (2013) 42:181–91. 10.1007/s10566-013-9196-z

[73] Garcia AM Sapyta JJ Moore PS Freeman JB Franklin ME March JS. Predictors and moderators of treatment outcome in the pediatric obsessive compulsive treatment study (POTS I). J Am Acad Child Adolesc Psychiatry. (2010) 49:1024–33; quiz 86. 10.1016/j.jaac.2010.06.013 20855047 PMC2943932

[74] Lavell CH Farrell LJ Waters AM Cadman J. Predictors of treatment response to group cognitive behavioural therapy for pediatric obsessive-compulsive disorder. Psychiatry Res. (2016) 245:186–93. 10.1016/j.psychres.2016.08.033 27544784

[75] Lewin AB Wu MS Murphy TK Storch EA. Sensory over-responsivity in pediatric obsessive compulsive disorder. J Psychopathol Behav Assess. (2014) 37:134–43. 10.1007/s10862-014-9442-1 PMC4504685 26190901

[76] Thompson-Hollands J Abramovitch A Tompson MC Barlow DH. A randomized clinical trial of a brief family intervention to reduce accommodation in obsessive-compulsive disorder: a preliminary study. Behav Ther. (2015) 46:218–29. 10.1016/j.beth.2014.11.001 25645170 PMC4748371

[77] Lebowitz ER Shimshoni Y. The SPACE program, a parent-based treatment for childhood and adolescent OCD- the case of jasmine. Bull Menninger Clin. (2018) 82:266–87. 10.1521/bumc.2018.82.4.266 30589579

[78] Paul D Askland KD Barlassina C Bellodi L Bienvenu Iii OJ Black D. Revealing the complex genetic architecture of obsessive-compulsive disorder using meta-analysis. Mol Psychiatry. (2018) 23:1181. 10.1038/mp.2017.154 28761083 PMC6660151

[79] Do Rosario-Campos MC Leckman JF Curi M Quatrano S Katsovitch L Miguel EC. A family study of early-onset obsessive-compulsive disorder. Am J Med Genet B. (2005) 136B:92–7. 10.1002/ajmg.b.30149 15892140

[80] Consortium C-DGotPG. Genomic relationships, novel loci, and pleiotropic mechanisms across eight psychiatric disorders. Cell. (2019) 179:1469–82. e11. 10.1007/s11682-019-00092-w 31835028 PMC7077032

[81] Sinopoli VM Erdman L Burton CL Easter P Rajendram R Baldwin G. Serotonin system gene variants and regional brain volume differences in pediatric OCD. Brain Imaging Behav. (2020) 14:1612–25. 10.1016/j.cell.2019.11.020 31187473 PMC10521965

[82] Mattheisen M Samuesl JF Wang Y Greenberg BD Fyer AJ McCracken JT. Supplementary information for “Genome-wide assocation study in obsessive-compulsive disorder: Results from the OCGAS”. Mol Psychiatry. (2014) 20:1–16. 10.1038/mp.2014.43 PMC4231023 24821223

[83] Walitza S Marinova Z Grunblatt E Lazic SE Remschmidt H Vloet TD. Trio study and meta-analysis support the association of genetic variation at the serotonin transporter with early-onset obsessive-compulsive disorder. Neurosci Lett. (2014) 580:100–3. 10.1016/j.neulet.2014.07.038 25093702 PMC4167890

[84] van Grootheest DS Cath DC Beekman AT Boomsma DI. Twin studies on obsessive-compulsive disorder: a review. Twin Res Hum Genet. (2005) 8:450–8. 10.1375/twin.8.5.450 16212834

[85] Pauls DL Abramovitch A Rauch SL Geller DA. Obsessive-compulsive disorder: an integrative genetic and neurobiological perspective. Nat Rev Neurosci. (2014) 15:410–24. 10.1038/nrn3746 24840803

[86] Cappi C Oliphant ME Peter Z Zai G Conceicao do Rosario M Sullivan CAW. De novo damaging dna coding mutations are associated with obsessive-compulsive disorder and overlap with tourette's disorder and autism. Biol Psychiatry. (2019) 87:1035–44. 10.1016/j.biopsych.2019.09.029 31771860 PMC7160031

[87] Lensi P Casssano G Correddu G Ravagli S Kunovack J Akiskal HS. Obsessive-compulsive disorder. Familial-developmental history, symptomatology, comorbidity and course with special reference to gender-related differences. Br J Psychiatry. (1996) 169:101–7. 10.1192/bjp.169.1.101 8818377

[88] Geller D Wieland N Carey K Vivas F Petty C Johnson J. Perinatal factors affecting expression of obsessive compulsive disorder in children and adolescents. J Child Adolesc Psychopharmacol. (2008) 18:373–9. 10.1089/cap.2007.0112 18759647 PMC2935829

[89] Vasconcelos M Sampaio A Hounie AG Akkerman F Curi M Lopes AC. Prenatal, perinatal, and postnatal risk factors in obsessive-compulsive disorder. Biol Psychiatry. (2007) 61:301–7. 10.1016/j.biopsych.2006.07.014 17123475

[90] Huppert JD Moser JS Gershuny BS Riggs DS Spokas M Filip J. The relationship between obsessive-compulsive and posttraumatic stress symptoms in clinical and non-clinical samples. J Anxiety Dis. (2005) 19:127–36. 10.1016/j.janxdis.2004.01.001 15488372

[91] Sasson Y Dekel S Nacasch N Chopra M Zinger Y Amital D. Posttraumatic obsessive-compulsive disorder: a case series. Psychiatry Res. (2005) 135:145–52. 10.1016/j.psychres.2004.05.026 15922457

[92] Gershuny BS Baer L Parker H Gentes EL Infield AL Jenike MA. Trauma and posttraumatic stress disorder in treatment-resistant obsessive-compulsive disorder. Depress Anxiety. (2008) 25:69–71. 10.1002/da.20284 17318836

[93] Lafleur DL Petty C Mancuso E McCarthy K Biederman Faro A Levy HC. Traumatic events and obsessive compulsive disorder in children and adolescents: is there a link? J Anxiety Disord. (2010) 25:513–9. 10.1016/j.janxdis.2010.12.005 21295942 PMC3074033

[94] McGregor NW Hemmings SM Erdman L Calmarza-Font I Stein DJ Lochner C. Modification of the association between early adversity and obsessive-compulsive disorder by polymorphisms in the MAOA, MAOB and COMT genes. Psychiatry Res. (2016) 246:527–32. 10.1016/j.psychres.2016.10.044 27821364

[95] Gorka SM Young CB Klumpp H Kennedy AE Francis J Ajilore O. Emotion-based brain mechanisms and predictors for SSRI and CBT treatment of anxiety and depression: a randomized trial. Neuropsychopharmacol. (2019) 44:1639–48. 10.1038/s41386-019-0407-7 31060042 PMC6785075

[96] Swedo S Leonard H Mittleman B Allen A Rapoport J Dow S. Identification of children with pediatric autoimmune neuropsychiatric disorders associated with streptococcal infections by a marker associated with rheumatic fever. Am J Psychiatry. (1997) 154:110–2. 10.1176/ajp.154.1.110 8988969

[97] Williams KA Swedo SE. Post-infectious autoimmune disorders: Sydenham's chorea, PANDAS and beyond. Brain Res. (2015) 1617:144–54. 10.1016/j.brainres.2014.09.071 25301689

[98] Mell LK Davis RL Owens D. Association between streptococcal infection and obsessive-compulsive disorder, Tourette's Syndrome, and tic disorder. J Pediatrics. (2005) 116:56–60. 10.1542/peds.2004-2058 15995031

[99] Kurlan R Johnson D Kaplan EL Group TSS. Streptococcal infection and exacerbations of childhood tics and obsessive-compulsive symptoms: a prospective blinded cohort study. Pediatrics. (2008) 121:1188–97. 10.1542/peds.2007-2657 18519489

[100] Tourette's Syndrome Study Group. Treatment of ADHD in children with tics: a randomized controlled trial. Neurology. (2002) 58:527–36. 10.1212/WNL.58.4.527 11865128

[101] Giedd JN Rapoport JL Garvey MA Perlmutter S Swedo SE. MRI assessment of children with obsessive-compulsive disorder or tics associated with streptococcal infection. Am J Psychiatry. (2000) 157:281–3. 10.1176/appi.ajp.157.2.281 10671403

[102] Singer HS. Autoantibody-associated movement disorders in children: proven and proposed. Semin Pediatr Neurol. (2017) 24:168–79. 10.1016/j.spen.2017.08.003 29103424

[103] Lepri G Rigante D Bellando Randone S Meini A Ferrari A Tarantino G. Clinical-serological characterization and treatment outcome of a large cohort of italian children with pediatric autoimmune neuropsychiatric disorder associated with streptococcal infection and pediatric acute neuropsychiatric syndrome. J Child Adolesc Psychopharmacol. (2019) 29:608–14. 10.1089/cap.2018.0151 31140830

[104] Frick LR Rapanelli M Jindachomthong K Grant P Leckman JF Swedo S. Differential binding of antibodies in PANDAS patients to cholinergic interneurons in the striatum. Brain Behav Immun. (2018) 69:304–11. 10.1016/j.bbi.2017.12.004 29233751 PMC5857467

[105] Attwells S Setiawan E Wilson AA Rusjan PM Mizrahi R Miler L. Inflammation in the neurocircuitry of obsessive-compulsive disorder. JAMA Psychiatry. (2017) 74:833–40. 10.1001/jamapsychiatry.2017.1567 28636705 PMC5710556

[106] Miller BJ Goldsmith DR. Inflammatory biomarkers in schizophrenia: Implications for heterogeneity and neurobiology. Biomark Neuropsychiatry. (2019) 1:100006. 10.1016/j.bionps.2019.100006

[107] Fullana MA Abramovitch A Via E Lopez-Sola C Goldberg X Reina N. Diagnostic biomarkers for obsessive-compulsive disorder: A reasonable quest or ignis fatuus? Neurosci Biobehav Rev. (2020) 118:504–13. 10.1016/j.neubiorev.2020.08.008 32866526

[108] Isung J Williams K Isomura K Gromark C Hesselmark E Lichtenstein P. Association of primary humoral immunodeficiencies with psychiatric disorders and suicidal behavior and the role of autoimmune diseases. JAMA Psychiatry. (2020) 77:1147–54. 10.1001/jamapsychiatry.2020.1260 32520326 PMC7287945

[109] Garvey M Perlmutter S Allen A Hamburer S Lougee L Leonard H. A pilot study of penicillin prophylaxis for neuropsychiatric exacerbations triggered by streptococcal infections. Biol Psychiatry. (1999) 45:1564–71. 10.1016/S0006-3223(99)00020-7 10376116

[110] Snider LA Lougee L Slattery M Grant P Swedo SE. Antibiotic prophylaxis with azithromycin or penicillin for childhood-onset neuropsychiatric disorders. Biol Psychiatry. (2005) 57:788–92. 10.1016/j.biopsych.2004.12.035 15820236

[111] Spartz EJ Freeman GM Jr. Brown K Farhadian B Thienemann M Frankovich J. Course of neuropsychiatric symptoms after introduction and removal of nonsteroidal anti-inflammatory drugs: a pediatric observational study. J Child Adolesc Psychopharmacol. (2017) 27:652–9. 10.1089/cap.2016.0179 28696783

[112] Nery FG Monkul ES Hatch JP Fonseca M Zunta-Soares GB Frey BN. Celecoxib as an adjunct in the treatment of depressive or mixed episodes of bipolar disorder: a double-blind, randomized, placebo-controlled study. Hum Psychopharmacol. (2008) 23:87–94. 10.1002/hup.912 18172906

[113] Muller N Schwarz MJ Dehning S Douhe A Cerovecki A Goldstein-Muller B. The cyclooxygenase-2 inhibitor celecoxib has therapeutic effects in major depression: results of a double-blind, randomized, placebo controlled, add-on pilot study to reboxetine. Mol Psychiatry. (2006) 11:680–4. 10.1038/sj.mp.4001805 16491133

[114] Shalbafan M Mohammadinejad P Shariat SV Alavi K Zeinoddini A Salehi M. Celecoxib as an adjuvant to fluvoxamine in moderate to severe obsessive-compulsive disorder: a double-blind, placebo-controlled, randomized trial. Pharmacopsychiatry. (2015) 48:136–40. 10.1055/s-0035-1549929 25959196

[115] Williams KA Swedo SE Farmer CA Grantz H Grant PJ D'Souza P. Randomized, controlled trial of intravenous immunoglobulin for pediatric autoimmune neuropsychiatric disorders associated with streptococcal infections. J Am Acad Child Adolesc Psychiatry. (2016) 55:860–7. e2. 10.1016/j.jaac.2016.06.017 27663941

[116] Martino D Schrag A Anastasiou Z Apter A Benaroya-Milstein N Buttiglione M. Association of group a streptococcus exposure and exacerbations of chronic tic disorders: a multinational prospective cohort study. Neurology. (2021) 96:e1680–93. 10.1212/WNL.0000000000011610 33568537 PMC8032367

[117] Mataix-Cols D Frans E Perez-Vigil A Kuja-Halkola R Gromark C Isomura K. A total-population multigenerational family clustering study of autoimmune diseases in obsessive-compulsive disorder and Tourette's/chronic tic disorders. Mol Psychiatry. (2018) 23:1652–8. 10.1038/mp.2017.215 29133949 PMC5951741

[118] Orlovska S Vestergaard C Bech B Nordentoft M Vestergaard M Benros M. Association of streptococcal throat infection with mental disorders: Testing key aspects of the pandas hypothesis in a nationwide study. JAMA Psychiatry. (2017) 74:740–6. 10.1001/jamapsychiatry.2017.0995 28538981 PMC5710247

[119] Kohler-Forsberg O Petersen L Gasse C Mortenson PB Dalsgaard S Yolken RH. A nationwide study in denmark of the association between treated infections and the subsequent risk of treated mental disorders in children and adolescents. JAMA Psychiatry. (2018) 76:271–9. 10.1001/jamapsychiatry.2018.3428 30516814 PMC6439826

[120] Murphy TK Patel PD McGuire JF Kennel A Mutch PJ Parker-Athill EC. Characterization of the pediatric acute-onset neuropsychiatric syndrome phenotype. J Am Acad Child Adolesc Psychiatry. (2015) 25:14–25. 10.1089/cap.2014.0062 25314221 PMC4340632

[121] Breithaupt L Kohler-Forsberg O Larsen JT Benros ME Thornton LM Bulik CM. Association of exposure to infections in childhood with risk of eating disorders in adolescent girls. JAMA Psychiatry. (2019) 76:800–9. 10.1001/jamapsychiatry.2019.0297 31017632 PMC6487907

[122] Dalmau J Gleichman AJ Hughes EG Rossi JE Peng X Lai M. Anti-NMDA-receptor encephalitis: case series and analysis of the effects of antibodies. Lancet Neurol. (2008) 7:1091–8. 10.1016/S1474-4422(08)70224-2 18851928 PMC2607118

[123] Gromark C Harris RA Wickstrom R Horne A Silverberg-Morse M Serlachius E. Establishing a pediatric acute-onset neuropsychiatric syndrome clinic: baseline clinical features of the pediatric acute-onset neuropsychiatric syndrome cohort at karolinska institutet. J Child Adolesc Psychopharmacol. (2019) 29:625–33. 10.1089/cap.2018.0127 31170007 PMC6786340

[124] Alexander GM Crutcher MD DeLong MR. Basal ganglia-thalamocortical circuits: Parallel substrates for motor, oculomotor, “prefrontal” and “limbic” functions. Prog Brain Res. (1990). 85:119–46. 10.1016/S0079-6123(08)62678-3 2094891

[125] de Wit S Alonso P Schweren L Mataix-Cols D Lochner C Menchón JM. Multicenter voxel-based morphometry mega-analysis of structural brain scans in obsessive-compulsive disorder. Am J Psychiatry. (2014) 171:340–9. 10.1176/appi.ajp.2013.13040574 24220667

[126] Weeland CJ White T Vriend C Muetzel RL Starreveld J Hillegers MHJ. Brain morphology associated with obsessive-compulsive symptoms in 2,551 children from the general population. J Am Acad Child Adolesc Psychiatry. (2021) 60:470–8. 10.1016/j.jaac.2020.03.012 32949714

[127] Milad M Rauch S. Obsessive-compulsive disorder: beyond segregated cortico-striatal pathways. TiCS. (2012) 16:43–51. 10.1016/j.tics.2011.11.003 22138231 PMC4955838

[128] Rasgon A Lee W Leibu E Laird A Glahn D Goodman W. Neural correlates of affective and non-affective cognition in obsessive compulsive disorder: A meta-analysis of functional imaging studies. Eur Psychiatry. (2017) 46:25–32. 10.1016/j.eurpsy.2017.08.001 28992533

[129] Sunol M Contreras-Rodríguez O Macià D Martínez-Vilavella G Martínez-Zalacaín I Subirà M. Brain structural correlates of subclinical obsessive-compulsive symptoms in healthy children. Eur Neuropsychopharmacol. (2017) 27:S1017–S8. 10.1016/S0924-977X(17)31783-2 29301668

[130] Huyser C Veltman DJ Wolters LH de Haan E Boer F. Functional magnetic resonance imaging during planning before and after cognitive-behavioral therapy in pediatric obsessive-compulsive disorder. J Am Acad Child Adolesc Psychiatry. (2010) 49:1238–48, 48. e1–5. 10.1016/j.jaac.2010.08.007 21093773

[131] Kalra SK Swedo SE. Children with obsessive-compulsive disorder: are they just “little adults”? J Clin Invest. (2009) 119:737–46. 10.1172/JCI37563 19339765 PMC2662563

[132] MacMaster F O'Neill J Rosenberg D. Brain imaging in pediatric obsessive-compulsive disorder. J Am Acad Child Adolesc Psychiatry. (2008) 47:1262–72. 10.1097/CHI.0b013e318185d2be 18827717 PMC2696312

[133] Boedhoe PSW Schmaal L Abe Y Alonso P Ameis SH Anticevic A. Cortical abnormalities associated with pediatric and adult obsessive-compulsive disorder: findings from the ENIGMA obsessive-compulsive disorder working group. Am J Psychiatry. (2018) 175:453–62. 10.1176/appi.ajp.2017.17050485 29377733 PMC7106947

[134] Kong X-Z Boedhoe PSW Denys D Van Den Heuvel OA Francks C. Mapping cortical and subcortical asymmetry in obsessive-compulsive disorder: findings from the ENIGMA consortium. Biol Psychiatry. (2020) 87:1022–34. 10.1016/j.biopsych.2019.04.022 31178097 PMC7094802

[135] Kooijman MN Kruithof CJ van Duijn CM Duijts L Franco OH van IMH. The Generation R Study: design and cohort update 2017. Eur J Epidemiol. (2016) 31:1243–64. 10.1007/s10654-016-0224-9 28070760 PMC5233749

[136] Gilbert AR Moore GJ Keshavan MS Paulson LAD Narula V MacMaster FP. Decrease in thalamic volumes of pediatric patients with obsessive-compulsive disorder who are taking paroxetine. Arch Gen Psychiatry. (2000) 57:449–56. 10.1001/archpsyc.57.5.449 10807485

[137] Jaspers-Fayer F Lin SY Chan E Ellwyn R Lim R Best J. Neural correlates of symptom provocation in pediatric obsessive-compulsive disorder. Neuroimage Clin. (2019) 24:102034. 10.1016/j.nicl.2019.102034 31734533 PMC6861668

[138] Fitzgerald KD Welsh RC Stern ER Angstadt M Hanna G Abelson J. Developmental alterations of frontal-striatal-thalamic connectivity in obsessive-compulsive disorder. J Am Acad Child Adolesc Psychiatry. (2011) 50:938–48.e3. 10.1016/j.jaac.2011.06.011 21871375 PMC3167379

[139] Geller DA Abramovitch A Mittelman A Stark A Ramsey K Cooperman A. Neurocognitive function in paediatric obsessive-compulsive disorder. World J Biol Psychiatry. (2018) 19:1–10. 10.1080/15622975.2017.1282173 28090807 PMC5555842

[140] Geller DA. Enduring effects of cognitive-behavioral therapy for pediatric obsessive-compulsive disorder: the nordic experience. J Am Acad Child Adolesc Psychiatry. (2017) 56:918–9. 10.1016/j.jaac.2017.09.417 29096772

[141] Abramovitch A Abramowitz JS Mittelman A. The neuropsychology of adult obsessive-compulsive disorder: a meta-analysis. Clin Psychology Rev. (2013) 33:1163–71. 10.1016/j.cpr.2013.09.004 24128603

[142] Bey K Kaufmann C Lennertz L Riesel A Klawohn J Heinzel S. Impaired planning in patients with obsessive-compulsive disorder and unaffected first-degree relatives: Evidence for a cognitive endophenotype. J Anxiety Disord. (2018) 57:24–30. 10.1016/j.janxdis.2018.05.009 29890378

[143] Bora E. Meta-analysis of neurocognitive deficits in unaffected relatives of obsessive-compulsive disorder (OCD): comparison with healthy controls and patients with OCD. Psychol Med. (2020) 50:1–10. 10.1017/S0033291720001634 32476632

[144] Geller D Biederman J Griffin S Jones J Lefkowitz TR. Comorbidity of juvenile obsessive-compulsive disorder with disruptive behavior disorders. J Am Acad Child Adolesc Psychiatry. (1996) 35:1637–46. 10.1097/00004583-199612000-00016 8973071

[145] Geller DA. The promise and challenge of obsessive-compulsive disorder research. Biol Psychiatry. (2007) 61:263–5. 10.1016/j.biopsych.2006.12.012 17241827

[146] Walitza S Zellmann H Irblich B Lange KW Tucha O Hemminger U. Children and adolescents with obsessive-compulsive disorder and comorbid attention-deficit/hyperactivity disorder: preliminary results of a prospective follow-up study. J Neural Transm. (2008) 115:187–90. 10.1007/s00702-007-0841-2 18200431

[147] Geller D March J Committee A. Practice parameter for the assessment and treatment of children and adolescents with obsessive-compulsive disorder. J Am Acad Child Adolesc Psychiatry. (2012) 51:98–113. 10.1016/j.jaac.2011.09.019 22176943

[148] Bridge J Iyengar S Salary CB Barbe RP Birmaher B Pincus HA. Clinical Response and risk for reported suicidal ideation and suicide attempts in pediatric antidepressant treatment: a meta-analysis of randomized controlled trials. JAMA. (2007) 297:1683–96. 10.1001/jama.297.15.1683 17440145

[149] Storch EA Wilhelm S Sprich S. Efficacy of augmentation of cognitive behavior therapy with weight-adjusted d-cycloserine vs placebo in pediatric obsessive-compulsive disorder: a randomized clinical trial. JAMA Psychiatry. (2016) 73:779–88. 10.1001/jamapsychiatry.2016.1128 27367832 PMC5734635

[150] Melin K Skarphedinsson G Thomsen PH Weidle B Torp NC Valderhaug R. Treatment gains are sustainable in pediatric obsessive-compulsive disorder: three-year follow-up from the NordLOTS. J Am Acad Child Adolesc Psychiatry. (2019) 59:244–53. 10.1016/j.jaac.2019.01.010 30768383

[151] Murphy TK Brennan EM Johnco C Parker-Athill EC Miladinovic B Storch EA. A Double-blind randomized placebo-controlled pilot study of azithromycin in youth with acute-onset obsessive-compulsive disorder. J Child Adolesc Psychopharmacol. (2017) 27:640–51. 10.1089/cap.2016.0190 28358599

[152] Storch EA Small BJ McGuire JF Murphy TK Wilhelm S Geller DA. Quality of life in children and youth with obsessive compulsive disorder. J Child Adolesc Psychopharmacol. (2017) 28:104–110. 10.1089/cap.2017.0091 28910139 PMC5831750

[153] Geller DA Biederman J Stewart SE Mullin B Martin A Spencer T. Which SSRI? a meta-analysis of pharmacotherapy trials in pediatric obsessive compulsive disorder. Am J Psychiatry. (2003) 160:1919–28. 10.1176/appi.ajp.160.11.1919 14594734

[154] March J Foa E Gammon P Chrisman A Curry J Fitzgerald D. Cognitive-behavior therapy, sertraline, and their combination for children and adolescents with obsessive-compulsive disorder: the pediatric ocd treatment study (POTS) randomized controlled trial. JAMA. (2004) 292:1969–76. 10.1001/jama.292.16.1969 15507582

[155] Højgaard D Hybel KA Ivarsson T Skarphedinsson G Becker Nissen J Weidle B. One-year outcome for responders of cognitive-behavioral therapy for pediatric obsessive-compulsive disorder. J Am Acad Child Adolesc Psychiatry. (2017) 56:940–7. 10.1016/j.jaac.2017.09.002 29096776

[156] Mancebo MC Boisseau CL Garnaat SL Eisen JL Greenberg BD Sibrava NJ. Long-term course of pediatric obsessive-compulsive disorder: 3 years of prospective follow-up. Compr Psychiatry. (2014) 55:1498–504. 10.1016/j.comppsych.2014.04.010 24952937 PMC4624317

[157] Eisen JL Sibrava NJ Boisseau CL Mancebo MC Stout RL Pinto A. Five-year course of obsessive-compulsive disorder: predictors of remission and relapse. J Clin Psychiatry. (2013) 74:233–9. 10.4088/JCP.12m07657 23561228 PMC3899346

[158] Fatori D de Braganca Pereira CA Asbahr FR Requena G Alvarenga PG de Mathis MA. Adaptive treatment strategies for children and adolescents with obsessive-compulsive disorder: a sequential multiple assignment randomized trial. J Anxiety Disord. (2018) 58:42–50. 10.1016/j.janxdis.2018.07.002 30025255

